# Invadosomes as “shape-shifters” of cellular maturation: insights from megakaryocytes

**DOI:** 10.3389/fcell.2025.1644011

**Published:** 2025-08-06

**Authors:** Frédérique Gaits-Iacovoni

**Affiliations:** Unité de Biologie Moléculaire, Cellulaire et du Développement (MCD, UMR 5077), Centre de Biologie Intégrative (CBI, FR 3743), Université de Toulouse, CNRS, UPS, Toulouse, France

**Keywords:** linear invadosomes, podosomes, cytoskeleton, extracellular matrix, metalloproteinase, CDC42 GTPase, collagen I, megakaryocytes

## Abstract

Invadosomes are a family of subcellular actin-based structures essential for cell–extracellular matrix (ECM) interaction and remodeling. In non-invasive cells, they are referred to as podosomes, which enable adhesion, migration, and ECM remodeling via secretion of metalloproteinases or mechano-traction. In invasive tumoral cells, these structures are called invadopodia due to their function. Despite structural similarity, podosomes appear as highly regular dots in 2D and do not always exhibit ECM-degradative abilities; hence, the term “degradative dot-podosomes” is used in this paper. Invadopodia are consistently degradative, fewer in number, slightly larger, deeper, less regular-shaped, and longer-lived. In tumor cells, collagen I induces the formation of linear invadosomes, which promote invasion by degrading collagen through the action of MT1–MMP (*membrane type 1*–*matrix metalloproteinase*) and the adaptor protein Tks5 (*tyrosine kinase substrate 5*). Interestingly, linear invadosomes also appear in non-tumor cells, such as megakaryocytes (MKs)—the platelet precursors—which display podosomes that closely resemble invadopodia. As MKs mature, Tks5 expression decreases, and dot-podosomes align along collagen I fibers, fusing into linear podosomes that remodel the ECM through mechanical traction but have lost their degradative ability. The GTPase Cdc42, crucial for invadosome formation, remains highly active in the MK internal demarcation membrane system (DMS) but is downregulated in linear podosomes. These observations suggest that Tks5, considered a marker of metastatic potential, also plays roles in normal physiology. Thus, linear podosomes with mechanotransductive properties may exist in a broader range of non-transformed cells. This mini-review focuses on the linear subfamily of invadosomes, highlighting their structure and function in MKs, a model in which invadosomes remain underexplored.

## Introduction

In all organisms, both physiological and pathological cells are surrounded by an extracellular matrix (ECM), composed of various proteins, whose stiffness depends on composition. Some ECMs tend to form soft gels, while others generate stiffer substrata, such as basement membranes around blood vessels or the osteoblasts near bone tissue, as examples. The most abundant ECM protein in the body is fibrillar collagen type I, which can form highly rigid bundles (Nilsson et al., 1998).

To cope with various ECM environments, cells have developed specialized subcellular structures at their ventral surface. These structures sense the ECM and transmit information to intracellular components via a central F-actin core and associated proteins, located at the center of invadosomes. This signaling modulates gene expression, as well as protein and lipid activity and dynamics, promoting cellular adaptation, ECM remodeling, or both. These structures belong to the invadosome family, which includes podosomes found in primary or cultured cells and invadopodia observed in transformed tumor cells with high metastatic potential (Cambi and Chavrier, 2021; Linder et al., 2023; Paterson and Courtneidge, 2018). Measuring 500 nm to 1 µM, both structures feature a central F-actin core that contains actin-remodeling proteins such as Arp2/3 (*actin-related protein 2/3 complex*), WASP (*Wiskott–Aldrich syndrome protein*), cortactin, the small GTPase Cdc42, and Tks5 (*tyrosine kinase substrate 5*), with these components being the most critical (Linder and Wiesner, 2015; Seals et al., 2005; Zagryazhskaya-Masson et al., 2020). The core is surrounded by a ring or cloud, which contains proteins linking the ECM to the intracellular cytoskeleton, via transmembrane receptors such as ß1 integrin, DDR1 (*discoidin domain receptor 1*), or CD44. These ECM receptors associate with mechano-sensitive proteins, vinculin, talin, or paxillin in the ring, to transmit forces into the cell cytoskeleton (Albiges-Rizo et al., 2009; Chabadel et al., 2007; Destaing et al., 2014; Destaing et al., 2010; Juin et al., 2014). Dorsal actomyosin fibers interconnect dot-podosomes through their caps, enabling coordinated movements and the formation of higher-order structures such as rosettes in endothelial cells or podosome belts, clusters, or sealing zones in osteoclasts (Luxenburg et al., 2007; Portes et al., 2022; Seano et al., 2014). Lateral actomyosin fiber contraction allows protrusions into the ECM (Linder and Wiesner, 2016).

Podosomes are found in a large variety of cells, including fibroblasts, monocytes/macrophages, dendritic cells, and sprouting endothelial cells (for review, see Linder, 2009; Linder et al., 2023). They also appear during development in neural crest cells and play essential roles in myoblast cell–cell fusion and megakaryocyte maturation in the bone marrow, enabling the release of mature platelets into the bloodstream (Eckly et al., 2020; Murphy et al., 2011; Sens et al., 2010). Thus, podosomes are involved in key physiological functions, such as adhesion, migration, and immune responses. Being frequently abundant, they appear in microscopy as regular circular structures; hence, the term dot-podosomes is used in this mini review to distinguish them from the linear forms currently under investigation by several groups (Linder et al., 2023; Paterson and Courtneidge, 2018).

Invadopodia are typically located exclusively beneath the nucleus and are fewer per cell than podosomes. They exhibit less regular morphology, appearing as irregular dot-like structures of varying widths and depth within the ECM. Although their core and ring components resemble those of podosomes, invadopodia are specifically associated with invasive and metastatic tumor cells, hence the term “invadopodia.” We now know that both dot-podosomes and invadopodia can secrete proteases, often MMP-2 or MMP-9, or, very commonly, MT1–MMP (*membrane type 1*–*matrix metalloproteinase*), to digest the ECM (Cecchetti et al., 2011; Ferrari et al., 2019; Malara et al., 2018; van den Dries et al., 2013). In this study, we define “degradative” dot- or linear-podosomes as those that express ECM-degrading proteins in non-tumor cells.

Invadosomes commonly express the scaffold protein Tks5 during formation and maturation. Tks5 binds phosphoinositides and various signaling proteins (e.g., Scr family kinases) involved in actomyosin regulation and, consequently, cytoskeleton dynamics. Both Cdc42 and Tks5 are critical for invadosome formation and have been widely described as required for tumor invasion and metastasis (Di Martino et al., 2014; Zagryazhskaya-Masson et al., 2020).

Invadosome formation occurs in several steps: (1) initiation, triggered by the ECM and growth factor signaling, leading to Cdc42 activation and the appearance of intracellular actin-rich dots; (2) maturation, involving the recruitment of actin-regulatory proteins, protein scaffolds, and lipids with signaling properties such as phosphoinositides, often through integrin/receptor-mediated signaling and Tks5 expression; (3) acquisition of proteolytic ability, mechanosensing or traction capacity, depending on ECM composition and the cell type involved; and (4) disassembly, a poorly understood process believed to involve actomyosin breakdown under RhoA GTPase control, will take place (Di Martino et al., 2016; Di Martino et al., 2014).

## Megakaryocyte and ECM interactions

Among the many cell types found in the body, megakaryocytes (MKs), the precursors of blood platelets, are of particular interest for studying cell signaling and cytoskeleton dynamics. Located in the bone marrow, MKs originate as small precursors in the osteoblastic niche and progressively differentiate into giant cells as they migrate toward the medullary sinusoids. Their maturation is a complex process involving endomitosis, the development of an internal membrane system known as the *demarcation membrane system* (DMS), and the synthesis of proteins required for future platelets. Once matured, MKs reach the vascular niche as large multinucleated cells (up to 24 N *in vivo* and 126 N *in vitro*), extending cytoplasmic elongations known as pro-platelets (PPTs) that unfold from the DMS. These projections cross the endothelial barrier to release platelets into the bloodstream (Italiano et al., 2007). This intricate maturation process is tightly regulated by cytokines and ECM components. Although the osteoblastic niche is enriched in fibrillar collagen I and fibronectin, the vascular niche is rich in collagen IV, laminin, and fibrinogen (Malara et al., 2015). As already mentioned, type I collagen is the most abundant ECM protein in the body, including the bone marrow. However, abnormal accumulation of collagen I fibers, as observed in myelofibrosis, can hinder cell motility, contributing to bone marrow failure and cytopenia. The interaction between MKs and their microenvironment is essential for triggering the cytoskeletal and membrane rearrangements required for proper differentiation. Like many other cells, MKs form invadosomes to interact with the ECM (Machlus et al., 2014).

## High-resolution imaging: a revolution in the study of podosome plasticity

The ability of MKs to form podosomes on fibronectin or fibrinogen was previously described (Schachtner et al., 2013), but their role in MK maturation remained poorly understood because of technical limitations in microscopy, along with the size and fragility of MKs. Interestingly, using high- or super-resolution microscopy, researchers observed the formation of F-actin lines along collagen I fibers. These structures resembled dot-podosomes in terms of protein composition but were morphologically similar to the linear invadopodia previously described in tumor cells (Ferrari et al., 2019; Juin et al., 2012). Our team and others have conducted in-depth investigations of invadosomes in MKs to better understand their nature and function compared to linear invadopodia in tumor cells. Advanced imaging techniques, including super-resolution photonic microscopy in cultured cells and whole bone marrow, along with correlated light and electron microscopy (CLEM), transmission electron microscopy (TEM), and focused ion beam/standard electron microscopy (FIB/SEM) on frozen bone marrow sections, have proven instrumental in understanding the relationship between invadosome structure and MK maturation, from progenitors cells to endothelium crossing and platelet production in the bloodstream (Eckly et al., 2020).

Recent advances have confirmed the presence of linear invadosomes with collagenase activity and Tks5 association in several cell types besides tumor cells (Aguilar et al., 2016; Eckly et al., 2020; Oprescu et al., 2022; Seano et al., 2014). In MKs, we found that linear podosomes form in response to collagen I stiffness. Our team was able to describe and record the alignment and fusion of dot-podosomes along collagen I bundles into linear F-actin-based structures, which were notably absent in progenitor cells (Oprescu et al., 2022). Interestingly, although dot and linear podosomes shared a majority of components in mature MKs (an F-actin core with cortactin, Arp2/3, and WASP, surrounded by talin, vinculin, and actomyosin), a striking difference appeared in progenitor dot-podosomes. They exhibited high levels of Tks5 and strong degradation activity, functionally resembling invadopodia. MK maturation seemed to be clearly associated with the maturation/function of invadosomes. As MKs matured, both Tks5 expression and ECM degradation capacity decreased, in parallel with DMS formation and endomitosis (Oprescu et al., 2022). These findings highlight that although Tks5 is a marker of ECM degradation capacity, it is not a reliable indicator of metastatic potential as it is also expressed in non-tumor cells under physiological conditions.

Interestingly, mature MKs use linear podosomes to exert traction on collagen I fibers, remodeling the ECM without degrading it, possibly to facilitate PPTs or large MK fragment passage through sinusoids. Unlike tumor cells, which rely on MT1–MMP for collagen degradation, MKs and progenitors secrete only MMP9, even though the presence of mRNA or proteins such as MMP1, MMP2, MMP9, and MT1–MMP has been reported (Cecchetti et al., 2011; Malara et al., 2018). Given the large size of mature MKs (>80 μm wide in suspension), it is reasonable to assume that they require ample space to migrate without damaging the marrow or blood vessels. Therefore, mechanical traction, rather than ECM digestion, appears to be a more tissue-preserving strategy for migration and transendothelial crossing.

## Structural remodeling of invadosomes in response to external cues

Our studies are among the first to describe in detail the linear restructuring and maturation of podosomes from dot-structures in MKs (Eckly et al., 2020; Oprescu et al., 2022). Most known podosome superstructures, such as rosettes or belts, are composed of clusters of individual dot-podosomes and do not involve full remodeling of the core and ring. In contrast, linear invadosomes lack many components of dot-podosomes (Juin et al., 2012; Linder et al., 2023). In MKs, the actin core fuses along the collagen fibers, and the surrounding ring proteins are reorganized around this new linear core. These components, including membrane receptors and cytoskeletal proteins, assemble into concentric layers that link collagen I fibers to intracellular F-actin, optimizing force transmission (Oprescu et al., 2022).

Interestingly, *in vivo*, MKs use a transcellular mechanism in which dot-like invadosomes located at the tips of PPTs or protrusions digest ECM components near the endothelium (e.g., collagen IV, which is abundant in this region). These structures may also function in surface sensing to identify ECM-free sites for transendothelial migration (Eckly et al., 2020). Evidence suggests that collagen I fibers could act as guiding tracks toward the sinusoids. We demonstrated that the transition from dot- to linear-podosome is reversible (Oprescu et al., 2022). Degradation activity could be reinitiated at PPT extremities upon contact with the endothelium or vascular niche, although further studies are needed to fully elucidate the molecular mechanism involved. It remains to be determined whether this reactivation involves the recruitment of Cdc42 and Tks5 to trigger MMP production and ECM degradation near the sinusoids. To explore this possibility, a co-culture model of endothelial cells and MKs is currently being developed to study invadosome dynamics and ECM remodeling in a reconstructed *in vitro* system. This approach is essential as current techniques do not yet offer sufficient resolution to visualize endogenous protein clusters *in vivo*.

Recent findings have shown that throughout megakaryopoiesis, Cdc42 remains associated with the DMS, where it displays strong activity (Antkowiak et al., 2024; Antkowiak et al., 2016; Dutting et al., 2017). In both primary MKs and the human UT711oc cell line differentiated with thrombopoietin (TPO), Cdc42 was detected along PPTs, as expected given its role in platelet activation. However, FRET-based measurements using probes derived from Raichu-Cdc42 (Viaud et al., 2014) revealed that Cdc42 activity is markedly lower in linear podosomes than in the DMS (Antkowiak et al., 2024). This finding supports the hypothesis that Cdc42 activity does not correlate with the degradative function of invadosomes. In MK progenitors, Cdc42 and Tks5 may function together or sequentially as part of a supramolecular complex that promotes invadosome formation and ECM digestion. In mature MKs, their activity appeared dispensable, in contrast to their essential role in highly invasive tumoral cells.

## Conclusion

**FIGURE 1:**
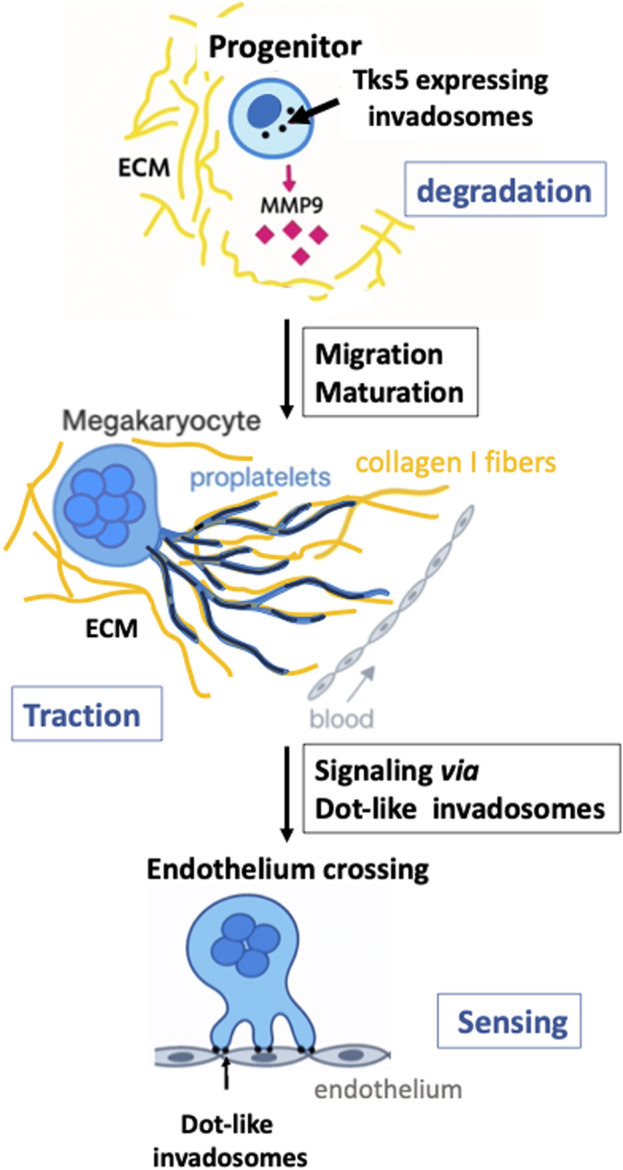
Changes in invadosomes during megakaryocyte maturation. Top panel: a small megakaryocyte progenitor displays multiple dot-invadosomes (black dots), which express Tks5 and secrete MMP9 (pink diamonds), enabling ECM degradation (depicted in yellow) and facilitating migration toward sinusoids. At this early stage of maturation, progenitors form degrading dot-podosomes. Middle panel: as MKs migrate toward the medullary sinusoids, they differentiate into large, multinucleated cells that have downregulated Tks5 and MMP9 expression. MKs then extend proplatelets (blue projections) toward the sinusoid. These proplatelets use collagen I fibers (long, dark yellow strands) as guidance tracks and form linear podosomes (black lines into the proplatelets) that elongate along the fibers. At this stage, ECM remodeling occurs *via* traction exerted by linear podosomes on collagen bundles. Bottom panel: when proplatelets and/or mature MKs reach the endothelium barrier of the sinusoids, contact is established through dot-like invadosomes capable of degrading the endothelial matrix (rich in collagen IV, fibronectin, and other components). These structures also function in surface sensing, initiating the formation of transendothelial pores for platelet release into the bloodstream (Eckly et al., 2020; Malara et al., 2014; Oprescu et al., 2022).

This mini-review highlights the relationship between structural changes and functional transitions occurring during the maturation of MKs, a physiological model in which invadosomes remain relatively unexplored (see Figure 1 for a schematic reconstitution). During the differentiation of progenitors into mature MKs, podosomes undergo structural, molecular, and functional remodeling in response to ECM and cytokine signaling. Proteins previously considered markers of invasive potential, such as Tks5, are dynamically regulated throughout MK maturation, in parallel with podosome organization (from dot-like to linear forms). This suggests a broader physiological role for Tks5 and emphasizes the plasticity of invadosomes beyond pathological contexts. Recent findings also point to a possible interplay between Tks5 expression and Cdc42 activity in the acquisition of mechanical properties that promote invadosome shape change, a feature that may be common to other cell types undergoing complex maturation programs. Further studies are needed to fully clarify this aspect.
